# Monthly Summary

**Published:** 1885-04

**Authors:** 


### Monthly Summary.

The First Permanent Molar.—*By J. H. Wooley.*—I am inclined to compare the first permanent molar to a waif, or outcast in the street, that is neglected through ignorance of its value. At the time when the first permanent molar is erupting, there are physical forces at work which disturb its harmonious growth. Neglect caused through ignorance of parents who think these teeth belong to the first set, plays a prominent part in their early destruction. If parents were familiar with the time these teeth usually erupt, and then placed their children in the hands of competent dentist, a large majority of the teeth could be saved. I believe it to be a mistake to remove these teeth for the purpose of correcting irregularity. Dr. Black says: "My study of the comparative liability of the teeth te decay at the different periods of life, shows clearly, that the first molars are attacked in the first two or three years after eruption, much oftener than any other teeth. But in after years they are oftener attacked less than the second molar. If the first and second molars are in a fair condition at the age of fifteen, the chances for the first are better than those of the second."

The coffer-dam should always be used in making an examination of these teeth; by this means we are better able to explore the decay. Often a slight decalcification is discovered, which can be stopped by a small filling, which, if neglected, will spread and soon involve the whole crown. My treatment of these teeth is as follows: In large cavities, where the inner surface is sensitive without pulp exposure, I fill with cement, allowing this to remain in the tooth from one to six months. I am careful first to flow over the floor of the cavity the cement in a soft condition, allow this to harden, then finish with the same material mixed very stiff. When this filling begins to show wear and the patient has had no trouble with the tooth, I partially remove the cement, leaving enough to cover the floor of the cavity, and am exceedingly careful that none of the cement is left clinging to the margins of the cavity. I then fill the remaining portion of the cavity with gold and tin combined, or whatever material seems best adapted to the case. If these teeth are taken in time, and carefully watched it will be, in most instances, the fault of the dentist, and not the teeth. if the cannot be saved.—*Ohio State Journal.*

---

REMOVAL OF THE DECIDUOUS TEETH.—*By Dr. O. A. Jarvis, New York.*—Nature has made provision for removal of the first set of teeth by a painless and healthy process of absorption of the roots. Almost without exception they will absorb fast enough to keep out of the way of the incoming teeth of the second set. Watchful parents are much troubled when they see the new tooth apparently crowded out of the proper line by the old one, and they want the old removed at once. This is *sometimes* necessary, but it is very seldom done except by ignorance or overpersuasion; and may result in serious and lasting injury to the patient. Even *crowding* during the growing age is accomplishing a purpose. It is positively necessary that the dentist should see the child often, and do with such teeth as he thinks best, for changes are rapidly taking place which may change the position of affairs favorably or unfavorably in a few weeks. When the natural process of absorption is not interferred with, the roots disappear, and the crown sits loosely on the gums, till it is easily removed with the fingers. This is not dying; there is no violence, disease or pain; it is simply passing peacefully away, as we ought to in good old age. But if from any cause (sometimes by accident, but generally by decay) they become dead and a source of irritation, they should be removed immediately. To retain them when nature is making such an effort to get rid of them, is unwise, and productive of much harm to the other parts.—*The Dentist.*

---

DEATH FROM NITROUS OXIDE GAS.—The occurrence of a death under nitrous oxide has lately caused a good deal of excitement in Paris. A retired magistrate, named Legeune, went to a M. Duchesne, a well-known advertising dentist of Paris, to have a tooth extracted. Gas was administered, and the operation performed; it was then discovered that the patient was dead. From the fact that there was no appearance of hemorrhage when the extraction took place, it is inferred that death must have occurred just prior to the operation· An examination of the body was made by Dr. Brouardel, but so far as we can learn, his report has not yet been made public. Judging, however, from the statements which appear in the French journals, death would seem to have been quite sudden, and to have been due to syncope, or failure of the heart's action, caused by the the fear or the shock of the operation. One result of this unfortunate occurrence has been, says the Editor of the *British Medical Journal*, that a dicussion has arisen in the French papers as to the right of dental practitioners to administer anæthetics. It appears that, as a matter of strict law, only legally qualified practitioners of medicine are allowed to administer anæsthetics in France, but that this law has seldom been put in force against dental practitioners, even with reference to the use of chloroform and ether. The use of nitrous oxide is not expressly forbidden; and, owing to the general impression that it was free from danger, no question has hitherto arisen as to its use. Whether any attempt will now be made to impose restrictions remains to be seen. Another point referred to in this correspondence is one of which we have repeatedly pointed out the importance, namely, the necessity for the presence of a third party, whenever an anæsthetic is administered, to give assistance in case of accidents, and also as a witness. Cases illustrating this point have so frequently come under our notice, and we have referred to the subject so often, that we hope it is unnecessary to say more.—*Items of Interest.*

